# Interplay of education and DNA methylation age on cognitive impairment: insights from the Health and Retirement Study

**DOI:** 10.1007/s11357-024-01356-0

**Published:** 2024-09-26

**Authors:** Erin B. Ware, César Higgins Tejera, Herong Wang, Sean Harris, Jonah D. Fisher, Kelly M. Bakulski

**Affiliations:** 1 Institute for Social Research, Survey Research Center , University of Michigan, 426 Thompson St, Ann Arbor, MI 48104 USA; 2 School of Public Health, Department of Epidemiology, University of Michigan, 1415 Washington Heights, Ann Arbor, MI 48109 USA; 3 School of Public Health, Department of Environmental Health Sciences, University of Michigan, 1415 Washington Heights, Ann Arbor, MI 48109 USA

**Keywords:** DNA methylation, Methylation clocks, Age acceleration, Dementia, Disparities

## Abstract

Few studies have assessed the association of educational attainment on dementia and cognitive impairment through DNA methylation age acceleration, while accommodating exposure-mediator interaction effects. We evaluated the mediation role of six epigenetic clocks with dementia, cognitive impairment non-dementia, and normal cognition, while accommodating exposure-mediator interaction effects. To understand the joint association of low education (≤12 years) and DNA methylation age acceleration (yes/no) in relation to cognitive impairment, we used weighted logistic regression, adjusting for chronological age, sex, race/ethnicity, and cell type composition. We performed four-way mediation and interaction decomposition analysis. Analyses were conducted on 2016 venous blood study participants from the Health and Retirement Study (*N* = 3724). Both GrimAge acceleration (OR = 1.6 95%CI 1.3–2.1) and low educational attainment (OR = 2.4 95%CI 1.9–3.0) were associated with higher odds of cognitive impairment in a mutually adjusted logistic model. We found additive interaction associations between low education and GrimAge acceleration on dementia. We observed that 6–8% of the association of education on dementia was mediated through GrimAge acceleration. While mediation effects were small, the portion of the association of education on dementia due to additive interaction with GrimAge acceleration was between 23.6 and 29.2%. These results support the interplay of social disadvantage and biological aging processes on impaired cognition.

**Supplementary Information:**

The online version contains supplementary material available at 10.1007/s11357-024-01356-0.

Alzheimer’s disease and its related dementias are neurodegenerative diseases where the loss of cognitive function severely affects one’s daily activities. Dementia affects 5.7 million Americans, with rising global prevalence due to an aging population structure [1]. Currently, dementias are incurable, and annual care is estimated at $236 billion [2]. Disparities in dementia are linked to structural and socioeconomic factors including racism and level of education [3–7]. Novel approaches characterizing the multifaceted etiology of dementia are needed to identify the biological underpinnings and potentially modifiable environmental factors that lead to dementia disparities.

Epigenetics—modifications to the genome that are not changes in DNA sequence—are potential indicators of adverse changes at a molecular level, uncovering risk of disease before the outcomes are observable. Epigenetic measures, such as DNA methylation, measured in brain tissue are associated with dementia [8–10]. DNA methylation patterns in peripheral tissues, such as blood, are also associated with dementia risk factors and dementia outcomes [11–13], providing a less invasive source for biomarkers compared to sources such as cerebrospinal fluid. These studies represent an important first step; however, foundational epigenetic epidemiology was primarily conducted in predominantly highly educated, white, and clinical dementia case-control samples. It is crucial that we now examine larger, more socioeconomically and racially diverse samples.

Epigenetic aging clocks are molecular biomarkers of aging based on patterns of DNA methylation at cytosine-phosphate-guanosine sites in the genome [14]. Chronologic clocks (e.g., Horvath and Hannum) were designed to predict chronological age based on DNA methylation sites [15, 16]. Phenotypic clocks (e.g., GrimAge and PhenoAge) built on the foundation of the chronologic clocks and incorporate additional factors to better reflect an individual’s health and aging process [17, 18]. Phenotypic clocks tend to be more strongly associated with aging phenotypes, like cognitive decline and Alzheimer’s disease than chronologic clocks [18, 19]. These DNA methylation clocks can be used to calculate age acceleration—or the difference between a chronologic and biological age. In fully understanding the efficacy of epigenetic clocks as a biomarker of dementia, it is critical to evaluate epigenetic clocks compared to chronological age and to evaluate how socioeconomic factors like educational attainment effect observed associations between epigenetic aging and dementia.

The relationship between DNA methylation, education, and cognition is complicated. We approach this complexity by using a mediation-interaction decomposition method [20]. A primary advantage over traditional mediation approaches, this mediation-interaction method allows us to decompose the total effect of education on cognitive impairment into four component parts: (1) the controlled direct effect of education, which indicates the association of education on cognitive impairment without age acceleration; (2) the interaction reference, which indicates the effect of the additive interaction that operates when age acceleration is present in those with more than 12 years of education, (3) the interaction mediation, which represents the additive interaction that operates only if education has an effect on age acceleration at different levels of educational attainment; and (4) the pure indirect effect, or the effect of age acceleration on cognition in those with >12 years of education. The purpose of this study was to evaluate the mediation-interaction of six different epigenetic clocks with education on the association with dementia and cognitive impairment compared to chronological age. We focus on three second-generation DNA methylation estimates (GrimAge, Dunedin pace of aging, and Levine) and include three chronological DNA methylation estimates (Horvath, Hannum, and Horvath-Skin). We also explored whether the epigenetic age acceleration mediates the association of education on cognitive impairment, while our mediation model accommodates for interaction effects between education and our epigenetic biomarker of interest. We used the Health and Retirement Study to investigate the relationship between epigenetic age acceleration (through epigenetic clocks) and impaired cognition among individuals with varying education status (≤12 years of education, >12 years).

## Methods

### Study population

The Health and Retirement Study is a nationally representative study sponsored by the National Institute on Aging (NIA U01AG009740). Surveys of adults over age 50 and their spouses in the USA are conducted by the University of Michigan, providing a national resource for data on changing health and economic circumstances associated with an aging population [21]. The Health and Retirement Study uses post-stratifying sampling weights methodology to obtain a representative sample of multiple birth cohorts of adults with diverse racial/ethnic backgrounds. Participants in the Health and Retirement Study provided written informed consent at the time of participation. Data collection procedures were approved by the University of Michigan Institutional Review Board. This secondary data analysis was approved by the University of Michigan Institutional Review Board (HUM00128220). All data used are publicly available through the Health and Retirement Study (https://hrs.isr.umich.edu/). Details on DNA methylation assays and DNA methylation quality control performed by the Health and Retirement Study are described in the Supplemental Materials.

### DNA methylation clocks and age acceleration

The Health and Retirement Study released epigenetic clocks calculated in R with coefficients posted alongside the accompanying manuscript of each epigenetic clock [22]. The clocks can be categorized as phenotypic (capturing aging-related outcomes) or chronologic (predicting traditional age). As our primary predictor, we prioritized the following phenotypic clocks: GrimAge, methylation pace of aging (DunedinPoAM38), and the Levine clock. The GrimAge clock incorporates biomarkers of physiological stress and DNA methylation based estimation of smoking pack-years to predict outcomes such as time to death, heart disease, and other age-related outcomes [17]. DunedinPoAM38 was developed using 18 biomarkers of rate of aging in various bodily systems (e.g., cardiovascular, renal, and pulmonary systems) [23]. The Levine clock, based on whole blood using 513 CpGs, predicts several clinical outcomes such as mortality, cancer, and physical function [18]. Sensitivity analyses include three other chronological epigenetic clocks, described in Supplemental Materials.

For each clock, epigenetic age acceleration (i.e., age accelerated residuals) was calculated using linear models and by regressing each DNA methylation clock (Y) on participant’s chronological age (X) at wave 2016. Residual values greater than zero reflected epigenetic age acceleration and residual values less than or equal to zero reflected epigenetic age at or less than chronological age. Age acceleration residual measures were dichotomized at the zero (the mean) for interpretability purposes. We considered continuous measure of age acceleration in sensitivity analyses.

### Cognitive status measures

We used a multidimensional measure of cognitive functioning based on a telephone-screening instrument: Telephone Interview for Cognitive Status [10]. Domains assessed using this measure include memory, mental status, abstract reasoning, fluid reasoning, vocabulary, dementia, and numeracy. In 2009, Langa, Kabeto, and Weir developed an approach for defining dementia and cognitively impaired non-dementia [24]. A team of dementia experts clinically validated this method using equipercentile equating against the Aging, Demographics, and Memory Study (ADAMS). The ADAMS study is a sub-sample of the Health and Retirement Study who received a more extensive neurological battery [25]. For self-respondents, the score consists of overall cognitive test performance. No proxy-rated respondents are included in these analyses since DNA methylation data were not collected in this group. All cognitive measures for these analyses were assessed in 2016 and taken from the HRS imputed cognition data set [26]. A score from 0 to 6 is categorized as dementia, 7 to 11 is categorized as cognitive impaired non-dementia, and 12 to 27 is categorized as normal cognition [24]. We explored sensitivity models using the Power’s dementia classification—an expert-defined algorithmic dementia categorization designed to reduce bias misclassification of dementia for racial disparities research [27].

### Covariate assessments

Self-reported educational attainment (years of school; categorized as “low education” for those with ≤12 years of education, and “high education” for those with >12 years of education), self-reported race/ethnicity (non-Hispanic White, non-Hispanic Black, and Hispanic), and sex (0 = female, 1 = male) were measured at a participant’s initial exam. Chronological age (years) was assessed at the 2016 exam. Cell type proportions were estimated from a complete blood count [22]. We included percent granulocytes and percent monocytes as precision variables in our models. Percent lymphocytes was not included, as the sum of percent granulocytes, monocytes, and lymphocytes was 100%.

### Four-level exposure variable

We created a four-level categorical variable consisting of all possible combinations between two dichotomized variables: education (≤ 12 years of education vs > 12 years of education) and epigenetic age acceleration (no age acceleration represented by an age acceleration residual value *≤* 0 vs accelerated aging with an age acceleration residual >0). Dichotomizing education at 12 years represents a less than high school education versus a high school graduate. This cutoff has been used in prior research involving the HRS and cognition (e.g., [28]). Our four-level primary predictor represents the joint association of epigenetic clock and education: a reference category representing the absence of both exposures (high education and age acceleration residuals ≤ 0); a second category denoting accelerated epigenetic aging (age acceleration residuals > 0 and high education); a third category denoting low education (≤ 12 years of education and age acceleration residuals ≤ 0); and a fourth category representing the presence of both exposures (≤ 12 years of education and age acceleration residuals > 0). While some loss of power is inherent in dichotomizing quantitative variables, we can better conceptualize and interpret the interaction of these factors as dichotomous variables [29, 30]. Further, the chosen cutoffs are meaningful for both educational attainment, representing the effect of a high school education, and epigenetic age acceleration, showing the effect for any age acceleration.

### Survey weights

We used survey weights for the Health and Retirement Study genetic sample from 2016 to generalize results to the Health and Retirement Study. We used these sample weights for descriptive statistics and all subsequent models.

### Statistical analysis

To properly account for the complex sampling design of the survey methodology and estimate weighted percentages, data were analyzed using the statistical software Stata 14 [31]. Participants were included with complete data on DNA methylation clocks, covariates, cognitive status, and sample weight variables. We described covariate distributions using mean and standard deviation for continuous covariates and number and frequency for categorical covariates. We compared the distributions of covariates among included and excluded participants using *t* tests for continuous covariates and chi-square tests for categorical covariates.

We calculated bivariate descriptive statistics on the combined sample and by cognitive status weighted by the 2016 genetic sample survey weights. We also tested the multivariable associations of each DNA methylation age acceleration method and education, adjusting for selected covariates, on either dementia or cognitive impairment non-dementia versus a reference category of normal cognition. These logistic models we weighted by the 2016 genetic sample survey weights.

**Fig. 1:**
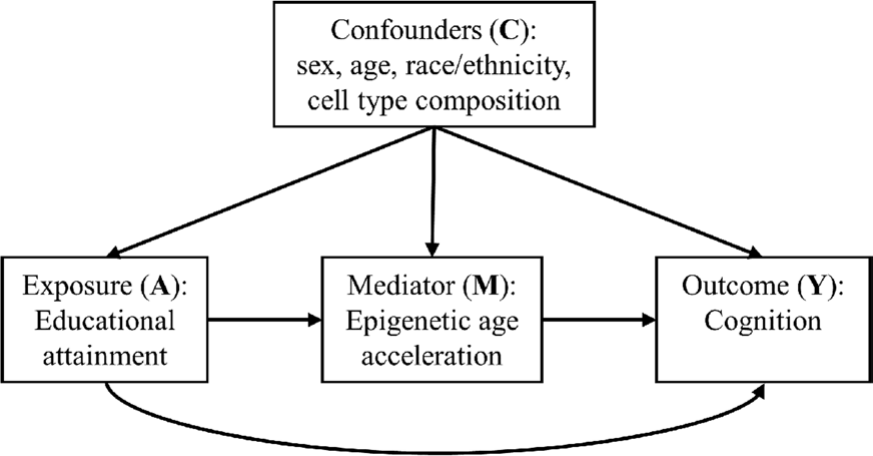
A heuristic model of our conceptual framework

We provide a conceptual model of our statistical analysis in Figure 1. We assessed additive interaction for all epigenetic clocks using impaired cognition as the outcome (i.e., dementia, cognitive impairment non-dementia, or Power’s dementia) relative to normal cognition, and we employed multivariable logistic regression to control for our main covariates of interest. Our base models included chronological age, self-reported race/ethnicity, sex, proportion granulocytes, proportion monocytes, and education as predictors. Subsequent models added dichotomized age acceleration residuals and an interaction between education and age acceleration. All models account for survey weights. We report odds ratios and 95% confidence intervals for interpreting effect estimates.

### Measures of interaction in the additive scale

We determined suitability to test for an additive interaction model of the joint association between an age acceleration measure (e.g., GrimAge acceleration, Levine age acceleration, etc.) and education if the sum of the odds ratio (OR) for the single exposed groups (OR_01_ + OR_10_, where OR_01_ is the odds ratio of DNA methylation age acceleration in those with educational attainment >12 years and OR_10_ is the association of educational attainment ≤12 in those with no DNA methylation age acceleration) was less than the odds ratio for the double exposed group (OR_11_, those with DNA methylation age acceleration and education ≤12 years). If these terms were satisfied, we would then calculate three measures of additive interaction for each age acceleration by education interaction: (1) the excess risk due to interaction, (2) the attributable proportion due to interaction, and (3) the synergy index. We used the delta method to calculate the corresponding 95% confidence intervals for these estimations [32]. In the context of our research, the excess risk due to interaction can be understood as the excess risk by which the interaction between age acceleration and $\le$ 12 years of education exceeds the addition of the isolated effects of both categories, that is, over and above their individual associations. The null value for the excess risk due to interaction is zero, a positive value is suggestive of synergism or interaction between the two variables and a negative value denotes antagonism. The attributable proportion due to interaction can be interpreted as the proportion of the excess risk in relation to cognitive impairment that is attributable to the interaction effect of epigenetic age acceleration and education $\le$ 12 years; the null value for this measure is also zero [32]. Finally, the synergy index represents the ratio between the joint association of age acceleration and $\le$ 12 years of education, divided by the isolated association of each of these two variables; a ratio of 1 represents the null value, and a ratio >1 is suggestive of superadditivity or synergism.

### Four-way mediation interaction decomposition analysis

After exploring the interaction effect between age acceleration and $\le$ 12 years of education, we sought to understand whether epigenetic age acceleration mediates the association of education on cognitive impairment, while our mediation model accommodated for potential interaction effects between exposure and mediator. We decomposed this mediation-interaction analysis using the four-way decomposition method to estimate (1) the controlled direct effect of education, which indicates the association of education on cognitive impairment without age acceleration; (2) the interaction reference, which indicates the effect of the additive interaction that operates when age acceleration is present in those with > 12 years of education [20]. We also estimated (3) the interaction mediation, which represents the additive interaction that operates only if education has an effect on age acceleration at different levels of educational attainment; and (4) the pure indirect effect, or the effect of age acceleration on cognition in those with >12 years of education. The sum of these four components equates to the total effect of educational attainment on cognitive impairment. We used these four measures to calculate the percentage attributable to each effect and employed the delta method to calculate associated confidence intervals for each estimation. Code to replicate these analyses is available at https://github.com/bakulskilab.

## Results

### Study sample descriptive statistics

**Fig. 2:**
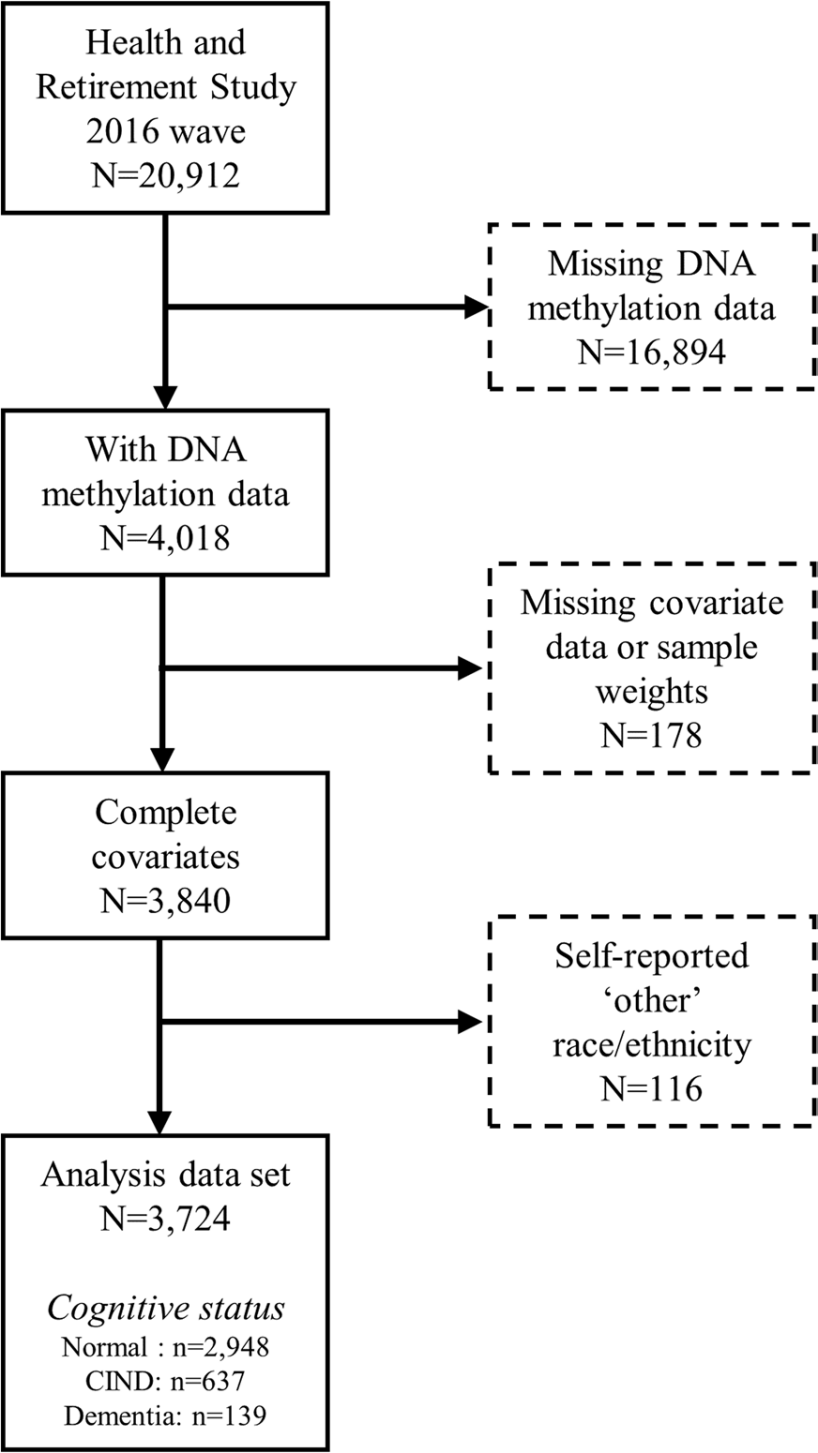
Analysis sample flow chart, Health and Retirement Study, 2016 wave. CIND: cognitively impaired, non-dementia

Of the 4018 participants with DNA methylation measures, 3724 had complete data for these analyses (Figure 2). Included and excluded participants were similar on most covariates, except self-reported race/ethnicity, sex, age, and cell type proportions (Supplemental Table 1). The analytic sample was predominantly non-Hispanic white (80.2%), female (53.9%), with an education >12 years (56.5%). The sample was 68.6 years on average. In bivariate descriptive statistics, the group with 12 or fewer years of education had higher weighted prevalence of dementia and cognitive impairment than the group with more than 12 years of education (Table 1).

**Table 1:** Weighted distribution of analytic sample covariates by cognitive status in the Health and Retirement Study, 2016 wave

	Overall *N* = 3724		Normal cognition *N* = 2948		Cognitive Impairment, non-dementia *N* = 637		Dementia *N* = 139	
	Col%	95%CI	Col%	95%CI	Col%	95%CI	Col %	95%CI
Race								
Non-Hispanic White (*n* = 2724)	80.2	[78.8,81.5]	83.7	[82.3,85.1]	65	[60.4,69.3]	49.7	[39.9,59.6]
Non-Hispanic Black (*n* = 628)	10.5	[9.5,11.6]	8.4	[7.4,9.5]	20.3	[16.7,24.5]	24	[17.0,32.7]
Hispanic (*n* = 526)	9.3	[8.4,10.4]	7.8	[6.8,9.0]	14.7	[11.9,18.0]	26.3	[18.4,36.1]
Sex								
Female (*n* =2149)	53.9	[51.9,55.9]	54.2	[52.0,56.5]	51.2	[46.4,56.0]	56.5	[46.5,66.0]
Male (*n* = 1575)	46.1	[44.1,48.1]	45.8	[43.5,48.0]	48.8	[44.0,53.6]	43.5	[34.0,53.5]
Education								
>12 years (*n* = 1853)	56.5	[54.5,58.4]	61.7	[59.6,63.8]	33.1	[28.7,37.9]	16.8	[10.2,26.5]
≤12 years (*n* = 1825)	43.5	[41.6,45.5]	38.3	[36.2,40.4]	66.9	[62.1,71.3]	83.2	[73.5,89.8]
Chronological age								
Age in years	68.6	[68.3,69.0]	67.5	[67.2,67.9]	73.4	[72.4,74.4]	77.6	[75.3,79.9]
Age accelerated residuals	Mean	95%CI	Mean	95%CI	Mean	95%CI	Mean	95%CI
GrimAge	−0.2	[−0.4, −0.1]	−0.6	[−0.8, −0.4]	1.4	[0.9,1.9]	1	[−0.1,2.0]
DunedinPoAM38	−0.4	[−0.6, −0.1]	−0.6	[−0.9, −0.4]	1	[0.4,1.6]	0.8	[−0.6,2.2]
Levine	0	[−0.3,0.3]	−0.2	[−0.5,0.1]	1.1	[0.4,1.8]	0	[−1.3,1.2]
Horvath	0	[−0.2,0.3]	0.1	[−0.2,0.4]	−0.2	[−0.8,0.5]	−0.4	[−1.8,0.9]
Hannum	0.1	[−0.1,0.3]	0.1	[−0.2,0.3]	0.5	[0.0,0.9]	−0.6	[−1.7,0.4]
Horvath—Skin	0	[−0.2,0.2]	0	[−0.2,0.2]	0.2	[−0.2,0.6]	−0.7	[−1.6,0.3]
Cell type proportions	Percent	95%CI	Percent	95%CI	Percent	95%CI	Percent	95%CI
Granulocytes	62	[61.6,62.3]	61.8	[61.4,62.2]	62.8	[61.8,63.8]	63.1	[61.1,65.1]
Monocytes	8.6	[8.5,8.7]	8.6	[8.5,8.7]	8.7	[8.4,8.9]	8.4	[7.8,8.9]
Lymphocytes	29.4	[29.1,29.8]	29.6	[29.2,30.0]	28.5	[27.6,29.4]	28.6	[26.7,30.4]

Note: Col%, Mean estimates and 95%CI represent statistics weighted using the 2016 genetic sample survey weights

### Main associations of low education and high epigenetic age acceleration with cognitive status

We show results for effect estimates of dichotomous education and age acceleration in mutually adjusted, weighted logistic models in Table 2. All models were adjusted for chronological age, sex, self-reported race/ethnicity, and cell type proportions and weighted by the 2016 genetic sample weights. Low education was significantly associated with dementia status (OR range 4.3–4.5, all *P* < 0.001) and also with cognitive impairment, non-dementia (OR range 2.4–2.6, all *P* < 0.001) (Table 2). High GrimAge acceleration was significantly associated with 1.63 times higher odds of cognitive impairment, non-dementia, relative to no age acceleration (95% CI, 1.3–2.1). No other dichotomous age acceleration variables were associated with dementia or cognitive impairment, non-dementia. We saw consistency in sensitivity models of continuous DNA methylation age acceleration (described in detail in Supplemental Materials and Supplemental Table 2).

**Table 2:** Weighted multivariable logistic regression results for mutually adjusted, education and dichotomized DNA methylation age acceleration residuals on dementia and cognitive impairment, non-dementia in the Health and Retirement Study, 2016 wave

Age acceleration calculation method^c^	Dementia vs normal cognition^a^		Cognitive impairment non-dementia vs normal cognition^b^	
	OR	95%CI	OR	95%CI
GrimAge				
No age acceleration	1	-	1	-
Age acceleration	1.49	[0.90,2.49]	1.63^***^	[1.28,2.08]
High education	1	-	1	-
Low education	4.32^***^	[2.40,7.77]	2.41^***^	[1.91,3.04]
DunedinPoAM38				
No age acceleration	1	-	1	-
Age acceleration	1.12	[0.67,1.88]	1.19	[0.93,1.52]
High education	1	-	1	-
Low education	4.45^***^	[2.49,7.95]	2.50^***^	[1.98,3.17]
Levine				
No age acceleration	1	-	1	-
Age acceleration	1.05	[0.68,1.64]	1.24	[0.98,1.56]
High education	1	-	1	-
Low education	4.49^***^	[2.51,8.05]	2.52^***^	[1.99,3.18]
Horvath				
No age acceleration	1	-	1	-
Age acceleration	1.04	[0.67,1.59]	0.92	[0.74,1.15]
High education	1	-	1	-
Low education	4.50^***^	[2.51,8.08]	2.55^***^	[2.01,3.22]
Hannum				
No age acceleration	1	-	1	-
Age acceleration	0.81	[0.51,1.29]	1.23	[0.98,1.55]
High education	1	-	1	-
Low education	4.51^***^	[2.51,8.10]	2.53^***^	[2.00,3.20]
Horvath—Skin				
No age acceleration	1	-	1	-
Age acceleration	0.98	[0.94,1.02]	1.09	[0.87,1.36]
High education	1	-	1	-
Low education	4.37^***^	[2.41,7.94]	2.54^***^	[2.01,3.21]

Exponentiated coefficients (odds-ratios); 95% confidence intervals in brackets

High education: > 12 years; low education ≤ 12 years

Age acceleration: >0 age acceleration residual; No age acceleration: *≤*0 age acceleration residual

^a^Dementia vs normal cognition (Langa-Weir classification), *n* = 3087

^b^Cognitive impairment, non-dementia vs normal cognition (Langa-Weir classification), *n* = 3585

^c^Models adjusted for sex, chronological age, self-reported race/ethnicity (non-Hispanic White used as reference group), percent of granulocytes, and percent of monocytes

^*^ *p* < 0.05, ^**^ *p* < 0.01, ^***^ *p* < 0.001

### Interaction effects in the additive scale

We observed statistically significant interactions using all methods of epigenetic clock calculations between no age acceleration and low education as well as age accelerated and low education on both dementia and cognitive impairment, non-dementia compared to normal cognition, with no age acceleration and high education as the reference group (all *P* < 0.05, Table 3). We additionally observed significant interactions between age acceleration and high education on cognitive impairment, non-dementia for both the GrimAge and DunedinPoAM38 epigenetic clock methods. For example, relative to the unexposed (those with no age acceleration and high education), participants with high GrimAge acceleration (and high education) had 1.5 times higher odds of dementia (95% CI, 0.4, 4.8), participants with low education (and no GrimAge acceleration) had 4.2 times higher odds of dementia (95% CI, 2.0–9.1), while participants with both low education and high GrimAge acceleration had 6.4 times higher odds of dementia (95% CI, 2.8–14.8).

**Table 3:** Weighted multivariable logistic regression additive interaction results for education and dichotomized DNA methylation age acceleration on dementia and impaired cognition, non-dementia in the Health and Retirement Study, 2016 wave

Age acceleration calculation method^c^	Dementia vs normal cognition^a^					Cognitive impairment, non-dementia vs normal cognition^b^				
	No age acceleration + High education (reference)	Age acceleration + High education	No age acceleration + Low Education	Age acceleration + Low Education	Additivity assumption satisfied?	No age acceleration + High education (reference)	Age acceleration + High education	No age acceleration + Low Education	Age acceleration + Low education	Additivity assumption satisfied?
	OR_00_	OR_01_	OR_10_	OR_11_	OR_11_ > OR_01_ + OR_10_	OR_00_	OR_01_	OR_10_	OR_11_	OR_11_ > OR_01_ + OR_10_
GrimAge										
	1	1.5	4.2^***^	6.4^***^	Y	1	2.0^***^	2.8^***^	4.1^***^	N
	-	[0.4,4.8]	[2.0,9.1]	[2.8,14.8]		-	[1.3,2.9]	[2.0,3.9]	[2.9,5.7]	
DunedinPoAM38										
	1	0.5	2.9^**^	4.0^***^	Y	1	1.5^*^	3.0^***^	3.1^***^	N
	-	[0.1,2.0]	[1.5,5.9]	[1.9,8.5]		-	[1.0,2.1]	[2.1,4.1]	[2.2,4.4]	
Levine										
	1	0.7	3.6^**^	4.2^***^	N	1	1.3	2.6^***^	3.1^***^	N
	-	[0.2,2.2]	[1.6,7.9]	[1.9,9.4]		-	[0.9,1.8]	[1.9,3.6]	[2.3,4.4]	
Horvath										
	1	1.7	6.6^***^	6.0^***^	N	1	0.9	2.5^***^	2.3^***^	N
	-	[0.5,5.6]	[2.5,17.0]	[2.3,15.8]		-	[0.6,1.3]	[1.8,3.4]	[1.7,3.2]	
Hannum										
	1	1.2	5.5^***^	4.1^**^	N	1	1.3	2.6^***^	3.1^***^	N
	-	[0.4,3.6]	[2.2,13.9]	[1.6,10.6]		-	[0.9,1.8]	[1.9,3.6]	[2.3,4.4]	
Horvath—Skin										
	1	0.5	3.2^**^	3.4^**^	N	1	1.2	2.7^***^	2.8^***^	N
	-	[0.2,1.4]	[1.4,7.0]	[1.5,7.5]		-	[0.8,1.7]	[2.0,3.8]	[2.0,3.9]	

Exponentiated coefficients (odds-ratios); 95% confidence intervals in brackets

High education: > 12 years; low education ≤ 12 years

^a^Dementia vs normal cognition (Langa-Weir classification), *n* = 3087

^b^Cognitive impairment, non-dementia vs normal cognition (Langa-Weir classification), *n* = 3585

^c^ Models adjusted for sex, chronological age, self-reported race/ethnicity (non-Hispanic White used as reference group), percent of granulocytes, and percent of monocytes

OR_01_ is the effect of high DNA methylation age acceleration in those with educational attainment >12 years and OR_10_ is the effect of educational attainment ≤12 in those with no DNA methylation age acceleration

^*^ *p* < 0.05, ^**^ *p* < 0.01, ^***^ *p* < 0.001

We determined if these associations were greater than additive by comparing the odds ratio in the double exposed group to the sum of the odds ratios in the single exposed groups. We observed greater than additive associations (OR_11_ > OR_01_ + OR_11_) in the models for GrimAge acceleration and the DunedinPoAM38 on dementia (Table 3). That is, for the GrimAge acceleration associations 6.4 > 1.5 + 4.2 and for the DunedinPoAM38 age acceleration residual associations 4.0 > 0.5 + 2.9 (Table 3). We performed four-way mediation-interaction effect decomposition on both these models.

The excess risk due to interaction between education and GrimAge acceleration was estimated to be 1.7 (95%CI, −1.2, 4.6), meaning the odds of dementia among participants with both exposures exceeds what one would expect if the association of age acceleration and low education were additive. Similarly, the attributable proportion was 0.3 (95%CI, −0.1, 0.6), suggesting that nearly 30% of the excess risk in the double exposed group can be explained by the interaction between the two exposures (low education and age acceleration). Finally, a synergy index of 1.5 (95%CI, 0.7, 2.7) indicates the presence of interaction between the two factors.

For DunedinPoAM38 acceleration, the excess risk due to interaction with education was estimated to be 1.5 (95%CI, −0.4, 3.5), meaning the odds of dementia among participants with both exposures exceeds what one would expect if the association of age acceleration and low education indicated additivity, though not significant. Similarly, the attributable proportion was 0.4 (95%CI, −0.0, 0.9), suggesting that nearly 40% of the excess risk in the double exposed group can be explained by the interaction between the two exposures (low education and age acceleration). Finally, a synergy index of 2.0 (95%CI, 0.7, 6.1) indicates the presence of interaction between the two factors. Of note is that for those with age acceleration and high education, the odds of dementia was less than one, though not significantly different from one. This may introduce a potential violation of the additivity assumptions.

### Four-way mediation-interaction effect decomposition

We performed a four-way effect decomposition analysis for education and age acceleration for the GrimAge and DunedinPoAM38 methods on dementia. We set continuous covariates at the mean (age, percent granulocytes, and percent monocytes) and sex as male. We separately estimated four-way mediation-interaction effects for each of the three self-reported race/ethnicities (non-Hispanic white, non-Hispanic Black, and Hispanic). For GrimAge acceleration, this mediation-interaction decomposition analysis suggests that most of the association of education on cognition is due to the controlled direct effect. The percent attributable to the controlled direct effect of education on dementia status was 74.6% (95%CI, 40.6–108.7) for the non-Hispanic White reference group, 69.5% (95%CI, 29.9–109.1) for the non-Hispanic Black group, and 74.4% (95%CI, 40.1–108.7) for the Hispanic group (Table 4). Additionally, we found that the percent of the association of education on cognition that is mediated through GrimAge acceleration is around 8.4% (95%CI, −1.1–17.9) for the non-Hispanic White group; 6.0% (95%CI, −0.2–12.2) for the non-Hispanic Black group; and 8.4% (95%CI, −1.0–17.7) for the Hispanic group. We calculated these estimates by adding the interaction mediation and the pure indirect effects. We found similar results for DunedinPoAM38 age acceleration in the percent attributable to the controlled direct effect of education on dementia status. The percent of the association of education on cognition that is mediated through DunedinPoAM38 acceleration is 5.0% (95%CI, −3.5–13.5) for the non-Hispanic White group, 3.6% (95%CI, −2.1–9.3) for the non-Hispanic Black group, and 4.8% (95%CI, −3.1–12.7) for the Hispanic group. Sensitivity models using the Power’s dementia classification can be found in Supplemental Materials and Supplemental Tables 2, 3, and 4.

**Table 4:** Four-way effect decomposition results for high education and no DNA methylation age acceleration based on the GrimAge and DunedinPoAM38 method on dementia in the Health and Retirement Study 2016 wave

	GrimAge				DunedinPoAM38			
	Non-Hispanic White				Non-Hispanic White			
	Excess effect on OR scale	95%CI	% attributable	95%CI	Excess effect on OR scale	95%CI	% attributable	95%CI
Controlled direct effect	3.2	[−0.0,6.5]	74.6%	[40.6,108.7]	1.9	[−0.1,4.0]	73.4%	[38.6,108.2]
Interaction reference	0.7	[−0.5,2.0]	17.0%	[−8.9,42.9]	0.6	[−0.2,1.3]	21.7%	[−1.8,16.4]
Interaction mediation	0.3	[−0.2,0.8]	6.6%	[−3.5,16.7]	0.2	[−0.1,0.4]	7.3%	[−1.8,16.4]
Pure indirect effect	0.1	[−0.2,0.4]	1.8%	[−4.3,7.9]	−0.1	[−0.1,0.0]	−2.3%	[−6.8,2.2]
Total	4.4	[0.4,8.3]			2.6	[0.4,4.9]		
Portion mediated			8.4%	[−1.1,17.9]			5.0%	[−3.5,13.5]
Portion due to interaction			23.6%	[−12.4,59.6]			28.9%	[−7.3,65.2]
	Non-Hispanic Black				Non-Hispanic Black			
	Excess effect on OR scale	95%CI	% attributable	95%CI	Excess effect on OR scale	95%CI	% attributable	95%CI
Controlled direct effect	3.2	[−0.0,6.5]	69.5%	[29.9, 109.1]	1.9	[−0.1,4.0]	63.6%	[23,104.2]
Interaction reference	1.1	[−0.8,3.1]	24.5%	[−10.1,59.1]	1.0	[−0.3,2.3]	32.8%	[−2.9,68.4]
Interaction mediation	0.2	[−0.2,0.6]	4.7%	[−1.9,11.3]	0.2	[−0.0,0.4]	5.3%	[−0.5,11.1]
Pure indirect effect	0.1	[−0.2,0.3]	1.3%	[−3.1,5.7]	−0.1	[−0.1,0.0]	−1.7%	[−4.9,1.5]
Total	4.7	[0.5,8.8]			3.0	[0.6,5.5]		
Portion mediated			6.0%	[−0.2,12.2]			3.6%	[−2.1,9.3]
Portion due to interaction			29.2%	[−12.0,70.4]			38.1%	[−3.3,79.5]
	Hispanic				Hispanic			
	Excess effect on OR scale	95%CI	% attributable	95%CI	Excess effect on OR scale	95%CI	% attributable	95%CI
Controlled direct effect	3.2	[−0.0,6.5]	74.4%	[40.1,108.7]	1.9	[−0.1,4.0]	69.9%	[32.6,107.2]
Interaction reference	0.8	[−0.6,2.1]	17.2%	[−9.0,43.5]	0.7	[−0.2,1.6]	25.3%	[−4.9,55.6]
Interaction mediation	0.3	[−0.2,0.8]	6.6%	[−3.4,16.6]	0.2	[−0.1,0.4]	7%	[−1.4,15.3]
Pure indirect effect	0.1	[−0.2,0.4]	1.8%	[−4.3,7.8]	−0.1	[−0.1,0.0]	−2.2%	[−6.5,2.1]
Total	4.4	[0.5,8.3]			2.8	[0.5,5.1]		
Portion mediated			8.4%	[−10,17.7]			4.8%	[−3.1,12.7]
Portion due to interaction			23.8%	[−12.4,60.1]			32.3%	[−6.3,70.9]

Models are adjusted (self-reported race/ethnicity as noted, male, of average age, average percent of granulocytes and monocytes) and weighted to be representative of the Health and Retirement Study in 2016

## Discussion

Inequities in early education can affect biological aging and perpetuate disparities in cognition later in life. Here, we characterized the relationships between epigenetic age acceleration measured in blood and educational attainment with dementia and cognitive impairment, non-dementia in a large, diverse sample. We observed a 1.6 times increase in the odds of cognitive impairment, non-dementia in those with DNA methylation age acceleration characterized using the GrimAge method relative to those with no DNA methylation age acceleration, after adjustment. Further, we saw a more than four times increase in the odds of dementia and a roughly 2.5 times increase in the odds of cognitive impairment, non-dementia in those with low educational attainment (≤12 years) compared to those with high educational attainment, after accounting for age acceleration. We observed interaction effects in the additive scale between low educational attainment and age acceleration using the GrimAge and DunedinPoAM38 methods on the odds of dementia. This includes a 6.4 times (95%CI, 2.8–14.8) higher odds of dementia in those with low education and high GrimAge acceleration and a 4 times (95%CI, 1.9–8.5) higher odds of dementia in those with low education and high DunedinPoAM38 age acceleration relative to the unexposed. We also observed that 6–8% of the proportion of the association of education on dementia is mediated through GrimAge acceleration, while 3.6–5% of the association of education on dementia may be mediated through DunedinPoAM38 age acceleration. In sensitivity analyses using the Power’s dementia algorithm to categorize dementia with greater attention to defining the outcome to elucidate racial disparities, we observed the association of education on cognition to be mediated at a much higher percent: 11–16.3%. Though there is need for additional research, these findings suggest the association of DNA methylation age acceleration using methods sensitive to phenotypes (i.e. GrimAge, Levine, and DunedinPoAM38) is small, but present with prevalent dementia. Our study further suggests DNA methylation age acceleration may interact with education in an additive manner in its association on dementia and impaired cognition.

To fully understand the efficacy of epigenetic age acceleration as a biomarker of dementia, it is critical to understand how socioeconomic factors including education modify observed associations between epigenetic aging and dementia across different race/ethnic populations. Epigenetic studies on minoritized social groups have been underpowered, and unfortunately, work on these same processes in Hispanic populations has been almost nonexistent—especially in nationally representative samples. Our study has taken steps to characterize associations between education, DNA methylation age acceleration, and cognition within groups racialized as non-Hispanic Black, non-Hispanic White, and Hispanic as well as examining a dementia outcome variable sensitive to race/ethnic disparities.

Our findings extend prior research linking biological aging and memory decline in later life from smaller, White European and New Zealand samples [18, 19, 23]. One recent study, using the same DNA methylation sub-sample of the Health and Retirement Study found that participants with lower SES—defined as a combination of education and wealth variables—had lower memory performance, faster decline, and exhibited accelerated biological aging (SES effect size associations (β) ranged from 0.08 to 0.41) [33]. This group further found that biological aging using DNA methylation accounted for 4–27% of the SES-memory gradient in White respondents, but there was minimal evidence of mediation in the Black or Latinx participants. This study examined three of the same epigenetic age acceleration variables as in our study: GrimAge, DunedinPoAM38, and the Levine methods. Consistent with their findings that accelerated biological aging was associated with lower memory performance, we did find an association between high GrimAge acceleration and cognitive impairment, non-dementia and between continuous DNA methylation age acceleration residuals and dementia, but we did not see associations between any of the other age acceleration methods and impaired cognition. Counter to their conclusion, though we did find some evidence for mediation, the percentage was small (6—8.4%) and the confidence intervals contained the null value of 0%. This value also did not appreciably change when using different racialized groups as reference categories.

Other studies have used different approaches to examine the mediating role of DNA methylation epigenetic aging between education and different phenotypes, specifically all-cause mortality. This group examined four of the same epigenetic clock biomarkers (Horvath, Hannum, Levine, and GrimAge) and calculated the proportion of educational inequalities in all-cause mortality explained by epigenetic aging biomarkers with a two-step analytic approach [34]. They first used a counterfactual-based mediation model and then a random-effects meta-analysis to pool estimates across cohorts. They further examined these relationships by sex. We did not investigate sex/gender differences within the context of race/ethnic groups due to sample size concerns. Like our conclusion, their evidence supports DNA methylation-based epigenetic aging as a signature of educational inequalities in the context of life expectancy, providing evidence that education and biological aging play a joint role in shaping health [34].

One potential limitation of our study is that DNA methylation was measured in venous blood samples. While brain tissue may appear to be the neurodegenerative disease epigenetic “gold standard”, the postmortem acquisition timing, scarcity of confounder/exposure data, and limited sample sizes with lack of replication potential present many limitations [35]. Postmortem samples may reflect epigenetic consequence of disease rather than cause; there may be bias in biological signals due competing causes of death [36], tissue Ph [37], or pre-mortem agonal state [38]. Peripheral blood with minimally invasive collection is the most abundant sample type for epidemiologic and clinical research. DNA methylation measured in peripheral tissues is associated with mortality [39–41], morbidity [14, 39, 41–44], and healthy aging [15, 18, 45, 46]. Importantly, peripheral DNA methylation is linked to impaired cognition [11–13], though much of this work has been on global or candidate gene DNA methylation. Epigenetic clocks representing accelerated aging have shown promise as potential biomarkers of dementia. Importantly, these age acceleration methods can be applied to DNA methylation obtained from whole blood, a relatively convenient and minimally invasive source for biomarkers. Our association is subject to reverse causation because the cross-sectional nature of this analysis prevents us from establishing that exposure and mediator precede outcome. Future studies should include assessments of reverse causation as well as other potential confounders or mediators, particularly health behavior and lifestyle factors. Lifestyle factors related to impaired cognition, physical activity and smoking for instance, may differ by both education and age acceleration. The selections of survivals and participant attrition prior to 2016 would introduce bias our result to null.

The effect sizes we observed for different methods of DNA methylation age acceleration varied by method and in significance. While these methods of characterizing age acceleration are potentially useful as biomarkers of later life cognition, DNA methylation is measured imprecisely, and these methods have varied reliability [47]. While many new and improved methods to assess DNA methylation biomarkers are being developed, our results only provide a beginning to the investigation of the utility of DNA methylation age acceleration, and its relationship to education and cognition and definitive conclusions based on these preliminary results should be avoided.

## Conclusion

Alzheimer’s disease and its related dementias remain prevalent health burdens that are unevenly distributed in the population across socioeconomic factors. It is imperative to identify conveniently obtainable biomarkers of dementia that can clarify the biologic underpinnings of dementia as well as modifiable environmental or socioeconomic factors that contribute to disparities in dementia risks. Our results demonstrate that age acceleration measured using DNA methylation clock methods differs in their associations with dementia and cognitive impairment status. Although additional research is needed, our results suggest that DNA methylation age acceleration does not substantively mediate the association between education and dementia or cognitive impairment. However, we do find a potential for additive interaction between education and DNA methylation age acceleration in our sample. Our findings highlight the need for more, large, multi-ethnic, population-based epigenetic studies of dementia that account for socioeconomic factors including education to better understand the interplay of social disadvantage and the biological aging process.

## Supplementary Information

Below is the link to the electronic supplementary material.

Supplementary file1 (DOCX 61 KB)

## Data Availability

All data used are publicly available through the Health and Retirement Study (https://hrs.isr.umich.edu/). Code to replicate these analyses is available at https://github.com/bakulskilab.

## Declarations

### Publication history

This manuscript was previously published in medRxiv; 10.1101/2022.12.23.22283919.
